# A multi‐laboratory assessment of lupus anticoagulant assays performed on the ACL TOP 50 family for harmonized testing in a large laboratory network

**DOI:** 10.1111/ijlh.13818

**Published:** 2022-03-01

**Authors:** Emmanuel J. Favaloro, Soma Mohammed, Ronny Vong, Kent Chapman, Priscilla Swanepoel, Geoffrey Kershaw, Nancy Cai, Sarah Just, Lynne Connelly, Timothy Brighton, Leonardo Pasalic

**Affiliations:** ^1^ Haematology Institute of Clinical Pathology and Medical Research (ICPMR) NSW Health Pathology Westmead Hospital Westmead New South Wales Australia; ^2^ Sydney Centres for Thrombosis and Haemostasis Westmead New South Wales Australia; ^3^ Faculty of Science and Health Charles Sturt University Wagga Wagga New South Wales Australia; ^4^ Haematology NSW Health Pathology John Hunter Hospital Newcastle New South Wales Australia; ^5^ Haematology NSW Health Pathology Prince Alfred Hospital Camperdown New South Wales Australia; ^6^ Haematology NSW Health Pathology Royal North Shore Hospital St Leonards New South Wales Australia; ^7^ Haematology NSW Health Pathology Prince of Wales Hospital Randwick New South Wales Australia; ^8^ University of Sydney Westmead New South Wales Australia

**Keywords:** activated partial thromboplastin time, lupus anticoagulant assays, Russell viper venom time, verification

## Abstract

**Introduction:**

Lupus anticoagulant (LA) testing is commonly performed within hemostasis laboratories, and the ACL TOP 50 family of instruments represent a new “single platform” of hemostasis instrumentation. Our aim was to evaluate these instruments and manufacturer reagents or alternatives for utility in LA testing.

**Methods:**

Comparative evaluations of LA testing using newly installed ACL TOPs 550 and 750 as well as comparative assessments with existing “reference,” predominantly Stago, instrumentation, and reagents. Evaluations comprised both dilute Russell viper venom time (dRVVT) and activated partial thromboplastin time (APTT)‐based assays. Establishment of normal reference ranges (NRR).

**Results:**

The HemosIL dRVVT‐based assays showed good comparability with the existing Stago reference method (R > 0.9) and could be considered as verified as fit for purpose. A variety of APTT assays was additionally evaluated for LA utility, and we identified from the assessment good utility of a non‐Werfen solution in Hyphen BioMed Cephen reagents. NRR were established based on ≥120 normal individual plasma samples.

**Conclusion:**

This evaluation of LA reagents on ACL TOP 50 Family instruments identified overall acceptable performance of both dRVVT (Werfen solution) and APTT (non‐Werfen solution) to enable harmonization of LA testing in our large network.

## 1 INTRODUCTION

Lupus anticoagulant (LA) reflects an acquired prothrombotic marker that comprises one laboratory criteria for establishing the presence of antiphospholipid (antibody) syndrome (APS).^1^, ^2^ LA testing also represents a common investigation in hemostasis/hematology laboratories^2^ and requires the performance of two tests based on different principles before LA can be excluded.^2^, ^3^ The latest guidelines from the International Society on Thrombosis and Haemostasis (ISTH) Scientific Standardisation Committee (SSC) on LA,^2^ in support of the previous guidelines,^3^ indicate that one of these test processes needs to be based on the dilute Russell viper venom time (dRVVT) as this method is very sensitive to LA. The RVVT is based on direct factor X activation by the snake venom.^4^, ^5^ The typical recommendation for the second method is a contact pathway assay, usually the activated partial thromboplastin time (APTT), which is based on activation of factor XII.^2^, ^3^, ^6^, ^7^, ^8^ The combination of RVVT and APTT for investigation of LA is also supported by CLSI (Clinical and Laboratory Standards Institute) guidelines on LA testing^7^ and is deemed sufficient for diagnosis or exclusion of LA, although some laboratories may undertake additional assays to further investigate such patients.^2^, ^3^, ^6^, ^7^, ^8^, ^9^, ^10^ For example, the silica clotting time (SCT) represents a sensitive contact activation pathway “alternative” to more classical APTT.^10^

In order to identify LA, testing is first performed with a screening reagent (i.e., dRVVT or APTT [or SCT]) that is considered sensitive or responsive to LA, and typically containing a low level of included phospholipids.^2^, ^3^, ^6^, ^7^, ^8^, ^10^, ^11^ If both test types provide clotting times within the normal reference range (NRR), then LA is excluded. Instead, a prolongation in test times (i.e., either or both dRVVT and/or APTT [or SCT]) is considered possibly suggestive of LA,^2^, ^3^, ^6^, ^7^, ^8^, ^10^, ^11^ although other causes of prolongation may alternatively be present, for example, anticoagulation therapy.^12^ Then, the test giving the prolonged test time is repeated using a confirmation reagent of the same assay type, but now containing a high level of phospholipid.^2^, ^3^, ^6^, ^7^, ^8^, ^10^, ^11^ This added phospholipid should swamp any LA present and thus yield a shortened clotting time compared to that of the screening test. This pattern is thus suggestive of LA. While there are several ways to measure this change in test times,^2^, ^3^, ^6^, ^7^, ^8^, ^10^, ^11^ in our geography the most common approach is by calculating the ratio of screen/confirm, primarily using results normalized to those of normal plasma in each test, to account for any inherent differences in clotting test times based on the reagents.^13^ Moreover, in most laboratories within our geography, a standard ratio cutoff of 1.2 is used to define presence of LA (≥1.2) or its absence (<1.2).^13^ Additional investigation by mixing studies is often recommended, although its position in the LA test algorithm may vary according to the LA guideline or expert opinion.^2^, ^3^, ^6^, ^7^, ^8^

The ACL TOP 50 family of instruments represent a collection of three hemostasis instruments comprising a “small” (350), an “intermediate” (550), and a “large” (750) model with similar features, but increasing throughput to facilitate hemostasis testing at sites with differing levels of complexities and needs. However, the family is treated as a single instrument class by both the manufacturer (e.g., common NRRs for assays across the platform) as well as by external quality assessment (EQA) organizations (including the main Australian provider, the RCPAQAP Haematology). We recently reported a major evaluation of routine coagulation tests (prothrombin time [PT], APTT, fibrinogen, thrombin time, D‐dimer) as performed on the three‐instrument platform, and which formed the basis for a 75 instrument rollout to 60 laboratory sites in our organization, New South Wales (NSW) Health Pathology (NSWHP), being the largest public pathology testing service in Australia.^14^ In this prior evaluation, we essentially confirmed the “equivalence” (or “interchangeability”) of all three TOP instrument types (350, 550, 750) in terms of assay results. More recently, we further reported an evaluation of congenital thrombophilia assays on this platform, using both manufacturer and alternate reagents.^15^ The current manuscript reports on a separate evaluation of the ACL TOP 50 family of instruments for LA assay testing in our network, to further enable standardization and harmonization of testing and procedures. We describe an evaluation of an LA assay manufactured by Instrumentation Laboratory (IL; being the manufacturer of the ACL TOP 50 family) and using their dRVVT method, as compared against existing equipment (i.e., Diagnostica Stago or Siemens Instrumentation) and existing dRVVT reagents (Stago) as “reference.” As IL does not supply an APTT‐based assay reagent pair for LA investigation, and since SCT is not as widely used in our geography, we further assessed a range of alternate APTT reagent options for utility in LA testing, as compared against existing methods as reference. This evaluation was also performed in order to satisfy our national accreditation (ISO 15189) standards and to provide a reference point as to any changes in test results to help guide our clients (clinicians and other pathology providers) through the changeover. We believe that the scope of our evaluation will be of contemporary interest and may assist other networks, both large and small, to achieve standardization and harmonization of specialized hemostasis tests on this instrument platform.

## 2 MATERIALS AND METHODS

### 2.1 Overview of setting and study design

This evaluation was undertaken by NSWHP personnel, and intended to eventually enable accredited implementation of identical methodologies in all NSWHP laboratories performing LA assays. Given LA testing is only performed within the larger sites of our organization, three sites (Table 1) partook in this evaluation. Specifically, the RVVT‐based LA assay as provided by IL was assessed against existing instrumentation and reagent in order to satisfy local National accreditation (ISO15189) requirements and to document any observed changes, which would be important for users of our service. A second evaluation was undertaken for evaluation of APTT‐based LA testing, and since IL do not provide a suitable reagent pair of LA sensitive/insensitive APTT reagents, and as SCT is not as widely used in our geography, we evaluated various APTT regents from both IL and alternate manufacturers/suppliers. This process is summarized in Figure S1. It should be noted that these evaluations supplement previous evaluations performed and as recently published by our group for routine coagulation tests^14^ and congenital thrombophilia testing^15^ performed of this instrument platform.

**TABLE 1:** Study sites participating in this evaluation^a^

Study site	Laboratory code	Instruments evaluated (ACL TOPs) (*n* = 3)	Existing instrument (Comparator) (Stago/Siemens)	Existing reagents (Comparators)
John Hunter Hospital (JHH), NSW Health Pathology, Newcastle, NSW	A	550	STAR Evolution	RVVT: Stago STACLOT DRRV Screen and Confirm APTT: Cephen and Cephen LS (Hyphen Biomed) Pooled normal plasma (PNP): Stago
Royal Prince Alfred Hospital, NSW Health Pathology, NSW	B	750	STAR Evolution	RVVT: Stago STACLOT DRRV Screen and Confirm APTT: Cephen and Cephen LS (Hyphen Biomed) PNP: Cryocheck (Precision Biologic)
Institute of Clinical Pathology and Medical Research (ICPMR), NSW Health Pathology, located at Westmead Hospital, NSW	C	750	STAR Evolution (APTT); Siemens CS 5100 (RVVT)	RVVT: Stago STACLOT DRRV Screen and Confirm APTT: Actin FS and FSL (Siemens) PNP: Stago

^a^ All sites located in Australia. All sites undertook testing for this evaluation, using the instruments (ACL TOPs) identified. All assays were performed as per manufacturer guidance. The same lots of IL dRVVT (DRVV screen N0192806, DRVV Confirm N0293700) and Cephan APTT (Cephen LS F1900691, Cephen (LR) F18000568P2) reagents were used at each site. For numbers of samples assessed at each site for each evaluation, please refer to each respective figure in Results.

The main instruments already in place at the three major evaluation sites were Stago STAR Evolutions (Table 1). An additional instrument from Siemens was utilized at one site (Table 1). The existing LA dRVVT‐based reagents were also from Stago (Table 1). The main variation in this evaluation was for APTT‐based LA reagents, where reagents were derived from a range of manufacturers (Table 1).

Each evaluation site contributed site‐specific data, using a moderate or large number of test samples and a wide range of test results covering both normal and pathological (i.e., LA) samples, as available for the evaluation. Samples used were those in excess to test needs, and after diagnostic testing according to the clinical request was completed. All samples were sodium citrate (3.2%) anticoagulated, centrifuged according to local validated centrifugation protocols. Some data related to identical or similar evaluations were also pooled to create a larger “composite” dataset.

### 2.2 Verification/establishment of normal reference ranges (NRRs)

We also performed several procedures to establish, or verify manufacturer recommended, NRRs for all assays as appropriate. For this purpose, we largely followed the CLSI guidance document “Defining, Establishing, and Verifying Reference Intervals in the Clinical Laboratory,”^16^ but also noted recommendations in some available LA guidelines.^2^, ^7^ For this evaluation, a number of normal donor individual plasma (INP) samples (healthy volunteers, non‐anticoagulated) were tested at several evaluation sites on the respective assays, and data was also combined for overall assessment. All samples were processed similarly to LA test samples, that is, double spun and frozen at −80°C prior to use, at which time they were thawed quickly in a 37°C water bath, gently mixed and tested like LA test samples. We assessed overall agreement based on the individual INP values, and also assessed means with ranges based on ±2 and ±3 standard deviations (SDs), as well as 2.5–97.5 percentiles and 99th percentile, also evaluating data for normality by several statistical methods.

### 2.3 Statistical analysis

We primarily evaluated comparative data by linear regression and Bland–Altman difference plots (GraphPad Prism software, La Jolla, CA, USA). As noted, some data were also assessed for normality using several statistical tools. Data are otherwise presented numerically or in qualitative synthesis.

### 2.4 Ethical considerations

According to guidance from local Human Research Ethics Committees, formal ethical approval for this evaluation was not sought, as the evaluation represents a Quality Assurance project of method verification using patient samples in excess to needs and which would otherwise be discarded after testing and mandatory short‐term storage according to local accreditation requirements.

## 3 RESULTS

### 3.1 RVVT‐based LA patient data

**FIGURE 1:**
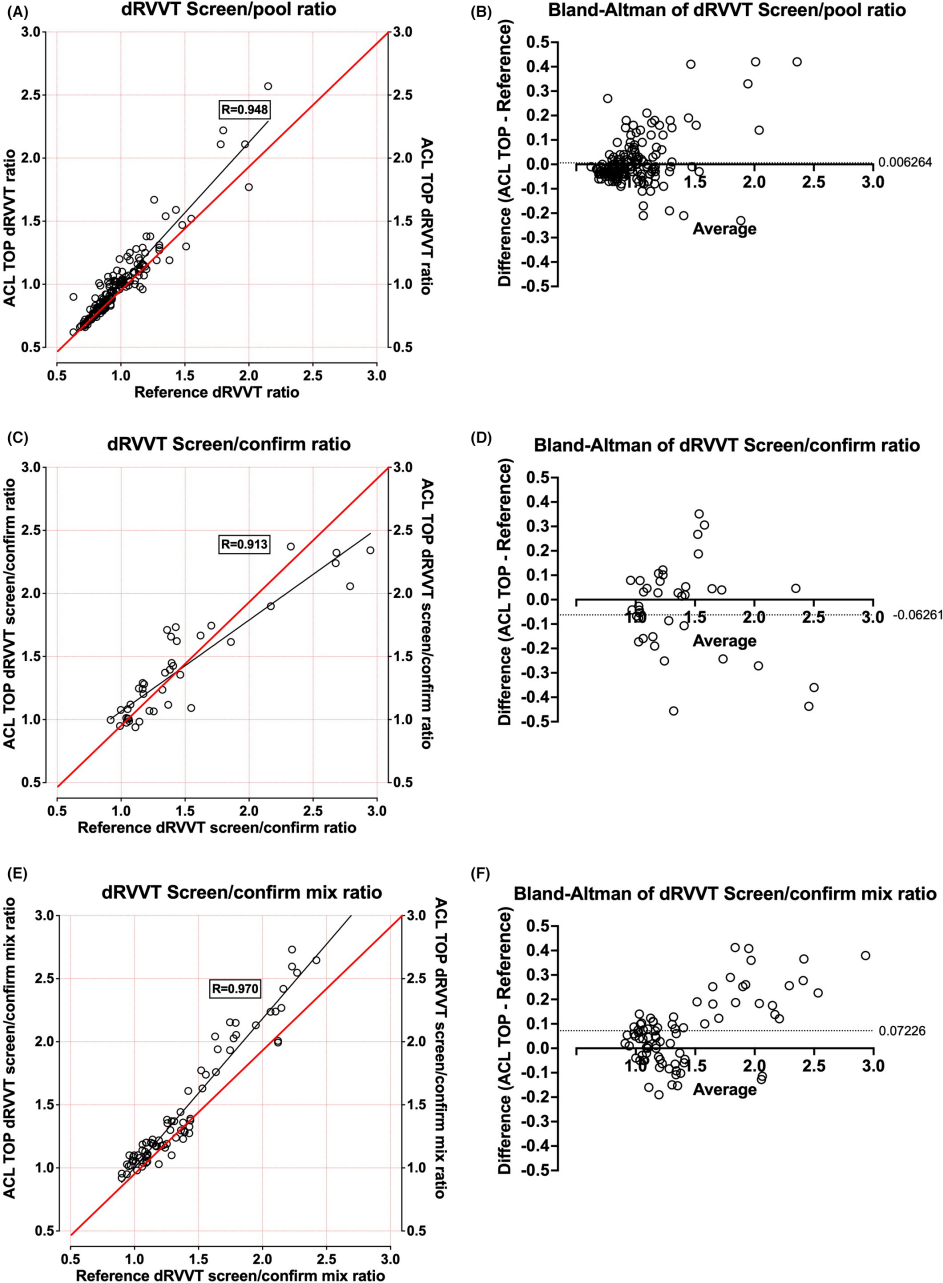
Comparative evaluation of lupus anticoagulant (LA) testing as performed by dilute Russell Viper Venom Time (dRVVT) assays on ACL TOP instruments using HemosIL reagents vs. Reference instruments using existing dRVVT (Stago) reagents—composite data. (A) Shows linear regression data for 176 samples co‐tested on ACL TOP vs. Stago analyser for dRVVT screen values reported as a normalized ratio against the pool normal plasma result with correlation coefficient (R) = 0.948. (B) Shows arising Bland–Altman difference plot for data in (A), with average bias = 0.006. (C) Shows linear regression data for 39 samples co‐tested on ACL TOP vs. existing analysers for dRVVT screen/confirm normalized ratios with correlation coefficient (R) = 0.913. (D) Shows arising Bland–Altman difference plot for data in (C), with average bias = −0.063. (E) Shows linear regression data for 86 samples co‐tested on ACL TOP vs. existing analysers for dRVVT screen/confirm mix normalized ratios with correlation coefficient (R) = 0.970. (F) Shows arising Bland–Altman difference plot for data in (E), with average bias = 0.072. See Figures S1–S3 for individual data on site‐based comparisons

Key composite data are shown in Figure 1, with additional site‐specific data shown in Figures [Link], [Link], [Link]. There was good correlation of HemosIL dRVVT screen assay reagent (normalized to normal pool value as a ratio) on ACL TOP vs. Stago dRVVT screen assay reagent on Stago analyser (Figure 1A; R = 0.948), with minimal bias (Figure 1B; average of 0.006). Similarly, there was good correlation of HemosIL dRVVT screen/confirm normalized ratios on ACL TOP vs. Stago dRVVT screen/confirm normalized ratios on existing analysers for both samples tested as neat plasma (Figure 1C; R = 0.913) and as 1:1 mix with normal plasma (Figure 1E; R = 0.970), again with small bias (respectively, Figure 1D and F). Some minor differences between reagents and instruments are to be expected and are also observed in EQA data (data not shown). However, there did appear to be a suggestion that the IL method was more LA sensitive on mix plasma testing than the reference methods, given slight‐positive bias (Figure 1F).

### 3.2 APTT‐based LA patient data

**FIGURE 2:**
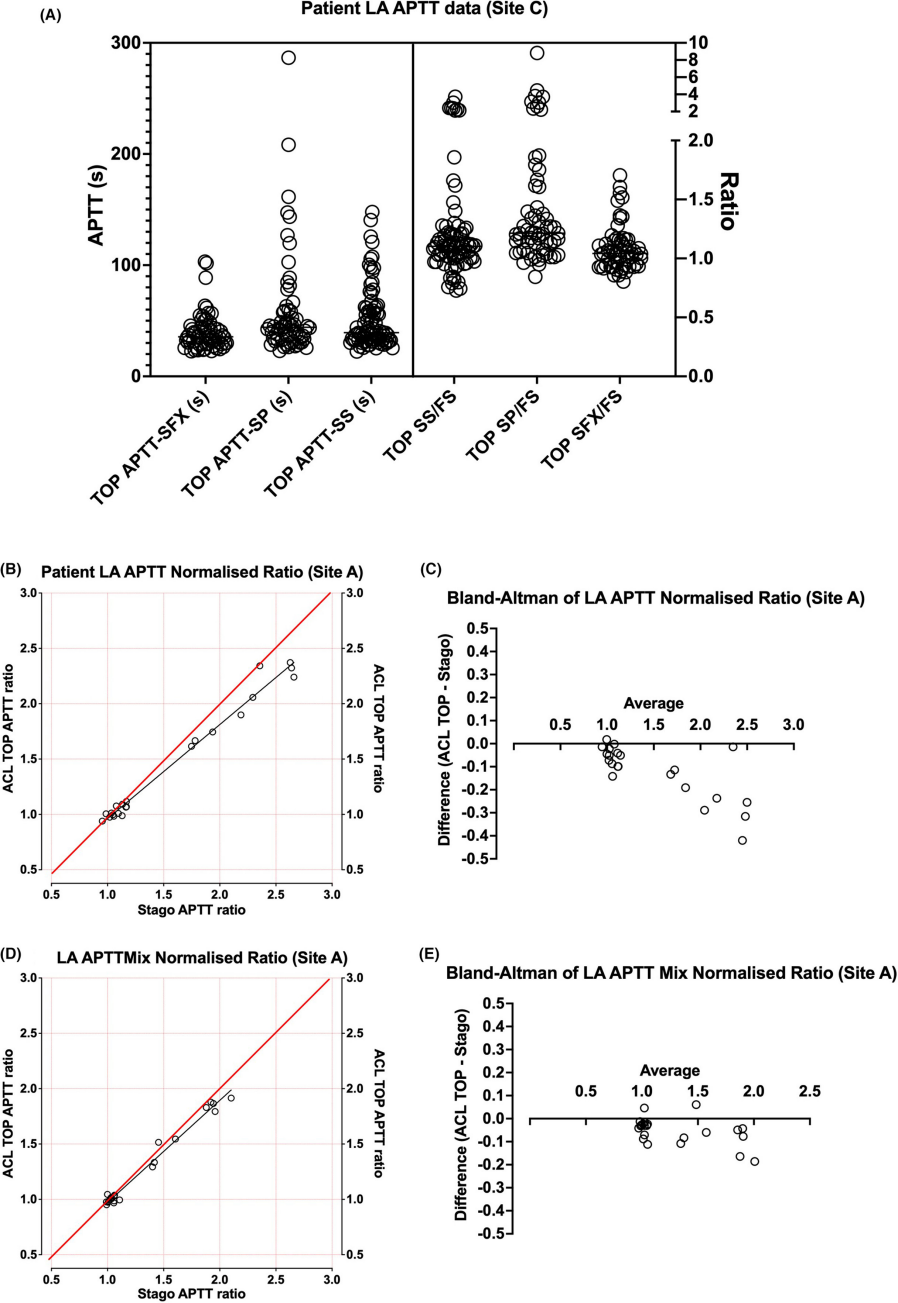
Comparative evaluation of APTT‐based LA testing using ACL TOP vs. Stago instruments using various APTT reagents—Site‐specific data. (A) Shows summary data for up to 81 samples for various APTT reagents with data shown as both clotting time (left y‐axis) and ratio against Siemens Actin FS reagent, as used at this site as the LA “insensitive” APTT reagent. Either IL SynthAFax (SFX) or SynthASil (SS) reagents were identified as capable of acting as a screen APTT in partnership with Actin FS. (B) Shows data from Site A, which evaluated their existing LA‐paired APTT reagents, Cephen LS and Cephen, respectively, as LA sensitive and LA insensitive, on ACL TOP vs. existing Stago instrument, using 21 samples. Data shown as a normalized ratio of Cepahen/Cephan LS. This identified essential agreement between LA‐positive and LA‐negative samples on either instrument platform, although perhaps with some negative bias shown on Bland–Altman analysis (C). Comparative data using 1:1 mix with normal plasma showed similar findings (D), but with less evident bias (E)

APTT patient‐based data for LA testing are shown in Figure 2, and showing site‐specific data since each site undertook different evaluations, according to pre‐existing practices at that site, and in order to share experiences and compare findings. Thus, site C undertook testing on a number of primarily IL APTT reagents using a number of LA test samples (Figure 2A). At this site, APTT‐based testing is performed on a Stago instrument using Actin FS as the LA insensitive reagent. Thus, the site chose to assess various IL APTT reagents in comparison with Actin FS and identified that both IL SP reagent and SynthASil reagent, but not SynthAFax, could provide options as LA sensitive reagents, if partnered with Actin FS. In contrast, Site A assessed Cephan APTT reagents on ACL TOP vs. existing Stago instrumentation and identified broad similarity (Figure 2B–E).

### 3.3 Establishment/verification of normal reference ranges (NRRs)

**FIGURE 3:**
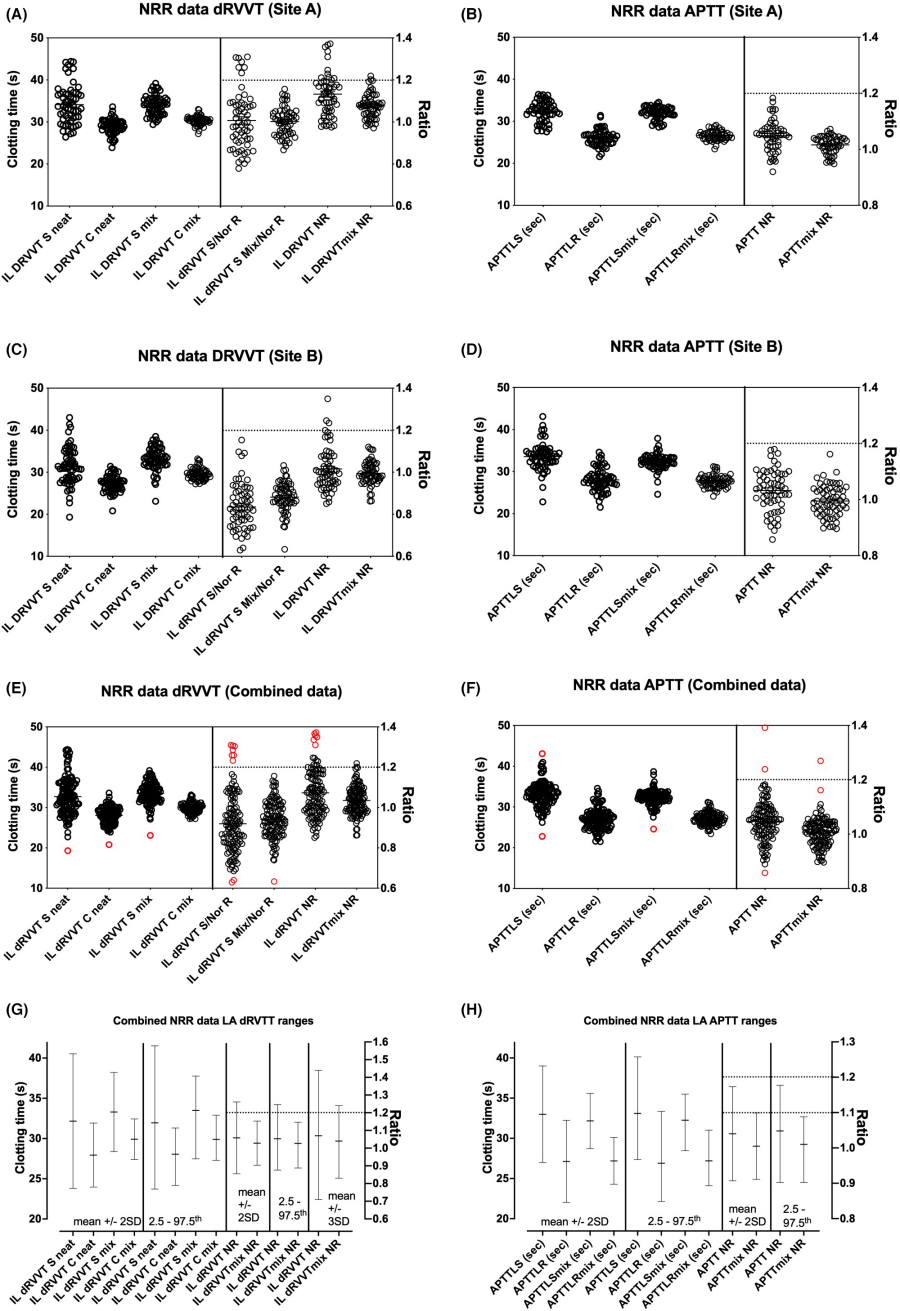
Summary data for establishing/verification of normal reference ranges (NRR) and values for LA‐negative/positive cutoffs. (A) (dRVVT) and (B) (APTT) show individual data points for 62 normal samples tested in Site A; (C) (dRVVT) and D (APTT) show individual data points for 60 normal samples tested in Site B; (E) (dRVVT) and (F) (APTT) show individual data points for 122 normal samples tested in Sites A and B as a composite. (G) (dRVVT) and (H) (APTT) show the NRR data shown as mean ± 2 SD or as 2.5–97.5th percentiles, as commonly performed depending on normality of data. NRR for dRVVT normalized screen/confirm ratio also shown for ±3 SD, as this statistical method is used by IL for their reported cutoff of 1.2. Red symbols indicate potential outlier data points that could be considered for removal for re‐evaluation of NRRs. Refer to Table 2 for numerical data summary and additional detail

Two sites were able to provided NRR data for both APTT‐ and dRVVT‐based testing on ACL TOPs, and essentially evaluating as per current practice at each site (Figure 3). Both sites A and B evaluated both dRVVT and APTT clotting times as both neat and 1:1 mix with normal plasma, as well as corresponding normalized ratios, as shown, respectively, in Figure 3A–D. For dRVVT, IL provides a manufacturer NRR for screen/confirm (based on 120 samples) with “low” (0.92) and “high” (1.11) ratio values based on their own normal test data distribution, as well as providing a recommended normalized screen/confirm LA cutoff of 1.2 (based on 40 samples and ±3 SDs). Site A and B data points closely fit within these limits for dRVVT, albeit a few values were above 1.2. However, given that >90% of INP samples yielded values below the 1.2 cutoff, for both individual site data (Figure 3A and C), as well as for combined data (Figure 3E), these ranges could be effectively verified as fit for purpose in our laboratories (for accreditation purposes) based on CLSI guidance.^16^ For APTT, Sites A and B again yielded similar data findings (Figure 3B and D), and also essentially confirmed a ratio of 1.2 as an effective upper cutoff ratio for LA “negativity” for accreditation purposes, especially when viewed as composite data (Figure 3F). We also assessed the normal ranges calculated for all assays and for normalized ratios, both as average ±2 SDs, or as 2.5–97.5th percentile as normally done for tests of hemostasis, depending on whether data is normally distributed or not, as well as assessing the average ±3 SDs as per the IL product insert, and also the 99th percentile, according to the latest ISTH SSC guidance.^2^ These data are shown in Figure 3G and H and summarized in Table 2. In brief, only some data passed multiple tests for normality (Table 2), and ranges defined by average ±2 SDs, or as 2.5–97.5th percentile were broadly similar (Figure 3G and H; Table 2). Instead, taking the average ±3 SDs or the 99th percentile led to a higher proposed cutoff closer to 1.3 or 1.4 than 1.2 for dRVVT screen/confirm normalized ratio (Table 2).

**TABLE 2:** Summary of normal reference range (NRR) data from current report^a^

	IL dRVVT S neat	IL dRVVT C neat	IL dRVVT S mix	IL dRVVT C mix	IL dRVVT S ratio	IL dRVVT S mix ratio	IL dRVVT S/C NR	IL dRVVT S/C mix NR	APTTLS (sec)	APTTLR (sec)	APTTLS mix (sec)	APTTLR mix (sec)	APTT NR	APTT mix NR
Number of values	122	122	122	120	122	122	122	120	120	121	120	121	120	121
Pass normality tests?	Some (2/4)	Yes (4/4)	Some (2/4)	Yes (4/4)	Some (2/4)	Yes (4/4)	No (0/4)	Yes (4/4)	No (1/4)	Yes (4/4)	No (0/4)	Yes (4/4)	Some (2/4)	No (0/4)
Median	32.1	28.2	33.6	30.0	0.90	0.94	1.06	1.04	33.2	26.9	32.3	27.2	1.05	1.01
2.5th −97.5th Percentile	**23.9–42.2**	**24.2–31.8**	**27.6–38.4**	**27.3–32.9**	**0.88–1.37**	**0.89–1.20**	**0.64–1.30**	**0.74–1.13**	**27.5–40.6**	**22.2–33.3**	**28.6–36.2**	**24.1–31.0**	**0.90–1.18**	**0.90–1.11**
99th Percentile	44.4	33.4	39.0	33.2	1.47	1.22	1.31	1.15	42.7	34.5	38.5	31.2	1.36	1.25
Mean	32.7	28.1	33.5	30.0	0.92	0.94	1.07	1.04	33.1	27.0	32.2	27.2	1.05	1.01
Range ±2 SD	23.2–42.2	24.0–32.1	28.3–38.7	27.4–32.5	0.61–1.23	0.76–1.13	0.83–1.32	0.90–1.17	26.9–39.3	21.9–32.2	28.6–35.9	24.2–30.1	0.90–1.20	0.90–1.11
Range ±3 SD	18.5–47.0	22.0–34.2	25.7–41.3	26.1–33.8	0.46–1.38	0.66–1.22	0.71–1.44	0.83–1.24	23.8–42.4	19.3–34.8	26.8–37.7	22.7–31.6	0.82–1.27	0.85–1.17

^a^ dRVVT S/C NR, dRVVT screen/confirm normalized ratio; dRVVT S/C mix NR, dRVVT mix screen/confirm normalized ratio (mix = 1:1 patient plasma:normal pool plasma). Data generated using data shown in Figure 3. No outliers were removed to create these ranges, which are primarily shown to identify the variation in ranges according to method used. Only few “outlier” data points (shown with red symbols in Figure 3) would be removed in first round by visual inspection, and thus such removal would not likely change ranges drastically. Of the statistical methods used, we would personally favor the 2.5th–97.5th ranges (bold text) as providing the most robust utility, given normal data was generally not normally distributed.

## 4 DISCUSSION

We present an evaluation of LA assays (RVVT and APTT based) on the ACL TOP 50 family of instruments (550 and 750 in this evaluation) as part of a verification process ahead of planned implementation and accreditation in all diagnostic laboratories that perform such testing and that form part of the largest Public Pathology test service in Australia, namely NSWHP.^14^ The overall evaluation has involved a number of separate evaluation exercises, as summarized in Figure S1, several APTT and dRVVT reagents, and several evaluation and comparator (“reference”) instruments at several evaluation sites. Each evaluation site is associated with tertiary level hospitals, thus having access to a wide variety of patient test samples, and each has extensive experience in laboratory reagent and equipment evaluations. The major evaluations were intended to facilitate accreditation to ISO 15189 standard and also fulfill other local National Accreditation requirements, and permit eventual standardization and harmonization of methods and procedures across all NSWHP laboratories. ISO 15189 standard requires comparison of the new proposed methods against existing methods used as reference, and our evaluation essentially confirmed the new IL dRVVT method performed on ACL TOP instruments to be “fit for purpose.” Accreditation further requires evidence of suitable peer comparison performance by EQA, which locally is provided by the RCPAQAP. Preliminary data are confirming suitable performance to date (data not shown). We are not aware of any similar multicenter evaluation process published in the literature. As already noted, the ACL TOP 50 Family of 350, 550, and 750 instruments are treated as a single instrument class by the manufacturer (same tests and detection methods, same NRR, etc.) as well as by the main EQA provider in Australia, the RCPAQAP. We also confirmed this three‐instrument “equivalence” in a previous evaluation study in relation to routine coagulation assays^14^ and also previously assessed the instruments and applicable reagents for congenital thrombophilia tests.^15^ The current evaluation also assessed IL methods/instruments/reagents against those already existing at the evaluation sites, but focussed on LA assays. Although primarily done to satisfy ISO 15189 and local National Accreditation standards, findings will also inform on any changes, and how best to advise clinicians who use our service in terms of prior or historical test result comparisons as required. We also established/verified manufacturer NRRs, with some minor adjustments for some tests, for example as based on numerical rounding, and as also supported by IL product information for dRVVT.

The evaluations at each site were sometimes similar and at other times different to those of other sites. This was because each site initially had independent separate processes for LA testing, albeit all being based on current guidelines.^2^, ^3^, ^7^ Indeed, the evaluation allowed the group to compare these different approaches and findings and to then agree on a path forward toward harmonization and standardization of test process at each site. Subsequent to the prior evaluations, and based on contractual arrangements, the group agreed on the use of IL SynthASil APTT reagent as the standard APTT reagent for use in the entire 75 instrument/60 laboratory network of NSW Health Pathology.^14^ Unfortunately, this reagent is LA sensitive, and IL does not manufacture an LA insensitive APTT reagent that can also provide good factor level sensitivity and also heparin sensitivity for use as a heparin therapeutic range.^14^ Moreover, although instead of APTT, IL provide SCT reagents for LA investigation, SCT procedures are not as widely employed in our geography, which might compromise the accreditation track. Given that IL do not manufacture an APTT LA‐paired reagent, the group therefore agreed to future use of the Cephen APTT reagent pair of Cephen LR and Cephen to provide the LA APTT test panel and thus enable group harmonization/standardization. This reagent pair reflects a good LA pair, is utilized by other RCPAQAP EQA participants, and would thus also enable effective peer assessment for accreditation purposes. In this respect, the current evaluation has identified acceptable NRR for these reagents, as well as the IL recommended 1.2 as an acceptable cutoff LA dRVVT screen/confirm normalized ratio for accreditation purposes (Figure 3; Table 2). Nevertheless, based on 2.5th–97.5th percentile, a cutoff of 1.3 would be statistically more correct (Table 2). The evaluation also identified comparability of this APTT reagent pair, as used on ACL TOPs, with existing “reference” equipment (Figure 2). For RVVT, the IL dRVVT pair of screen and confirm reagents was also seen as largely comparable to those in current use (Figure 1). Moreover, NRR data for dRVVT were similar between sites and compatible with ranges provided by the manufacturer (Figure 3).^16^ If we were to follow the ISTH SSC guidance, which recommends a 99th percentile cutoff, then the cutoff would need to be 1.3 (Table 2) for testing using neat plasma, with 1.2 being acceptable when testing is performed as mixing studies. The LA guidance from CLSI, however, tends to argue against use of the 99th percentile approach, given the multiple steps in the LA process.^7^

Some evaluations of ACL TOP instruments have been previously published, as also previously reported by us,^14^, ^15^ but none of those previous reports discussed evaluation of LA assays on ACL TOPs, as did none of the cited literature, which was largely focused on performance of the pre‐analytical module for detection of hemolysis/icteria/lipemia.^14^ Our review of the literature in relation to evaluations of LA assays on ACL TOPs only uncovered few prior evaluations, none of which were comparable to ours. For example, Kanouchi et al undertook an evaluation of LA by APTT using waveform analysis on an ACL TOP analyser to investigate patients with vs. without thrombosis.^17^ The authors utilized IL APTT‐SP reagent. Seheult et al assessed for spectral interference in a range of optical end‐point coagulation assays on an ACL TOP 750 analyser and including LA testing.^18^ They identified that hemolysis indices up to 900 mg/dL did not affect the APTT or dRVVT confirm, but that indices above approximately 200 mg/dL resulted in a false‐negative dRVVT screen and screen/confirm ratio in samples with a LA. Esmedere Eren et al evaluated APTT clot derivative curves, as available on ACL TOPs, and using SythASil APTT reagent, and concluded they had utility in the investigation of LA patients.^19^ Falay et al utilized an ACL TOP analyser to evaluate 166 patients with an isolated raised APTT, and utilized SythASil APTT reagent in combination with repeat testing by Stago Cephascreen APTT on a Stago STAR analyser.^20^ They showed correction upon repeat testing in nearly half the samples, while of those still prolonged, following mixing studies, LA or factor inhibitors could, respectively, be identified in four patients each.

There are also very few multicenter studies looking at LA testing, and none that we could locate that was comparable to ours. For example, Moore and colleagues have recently reported a multicenter study centered on Taipan snake venom time as a LA screening test with ecarin time as the confirmatory test.^9^ The outcome of this study was the validation of these assays LA detection in non‐anticoagulated patients and in those on vitamin K antagonists (VKAs) or direct factor Xa inhibitors. Sciascia and coworkers^21^ assessed the reliability of LA testing in a multicenter setting, to identify a concerning 45% discrepancy in results for LA, despite increasing international standardization initiatives such as by the ISTH SSC.^2^, ^3^ However, this was based on a cohort of 60 patients that fulfilled their specified inclusion criteria, which included also patients on anticoagulation therapy. Of course, variation in LA testing and test results is well known in this field, including evidence from the EQA setting,^13^ and depends on the tests and procedures used by laboratories, which was also evident to some extent with the group involved in the current assessment in regard to prior LA‐based APTT testing (Table 1). Moreover, the current report aims to help standardize future LA testing to reduce such potential variability in the future. Along these lines, Tripodi et al have recently investigated the variability of LA cutoff values.^22^ These were calculated as dRVVT screen/PNP (pooled normal plasma) ratios in 11 laboratories, each testing plasma from 120 donors with 3 platforms. They observed major variation, even within the same platform. They also reported differences between cutoff values calculated as 99th or 95th centiles that translated into a different LA detection rate (i.e., the lower the centile the greater the detection rate). The take‐home message from this study was that cutoff values determined as the 95th centile allowed a better LA detection rate. None of the included laboratories utilized the Cephen APTT reagents, as included in our study, but 4/11 utilized IL dRVVT reagents on ACL TOP instruments (2x ACL TOP 500, 2x ACL TOP 700; but no members of the ACL TOP 50 family of instruments). Thus, their study provides a partial comparator. The median dRVVT screen/PNP ratio obtained using IL dRVVT and ACL TOPs (500, 700) as a 95th percentile ranged from 1.10 to 1.22, and for the 99th percentile ranged from 1.27 to 1.56. Another potentially relevant study, we could identify is by Poz et al.,^23^ who reported a multicenter study for a standardized and harmonized reporting of LA. They processed 100 normal samples, 20 each from 5 centers, to confirm negative upper limits and calculate positivity cutoffs of LA integrated assays including dRVVT. They also utilized 311 samples previously identified by the laboratories as being positive for LA to characterize different positivity levels for each assay. For healthy subjects, the negative upper limit was set at 1.17 for dRVVT normalized ratio based on 99th percentile, and their positivity cutoff was set at 1.20 based on added CV%. Their study was also based on the IL dRVVT reagents, and all centers employed ACL TOP 700 instruments. Corresponding values for SCT were 1.19 (negative upper limit) and 1.23 (positivity cutoff). These values were eventually adopted by all 5 centers in their aim for local standardization. In summary, there is recognized variability in the cutoffs identified by different studies, even when laboratories use the same method, and also due to differential selection of 95th or 99th percentile.

We acknowledge several limitations in our study. This study was a laboratory‐based evaluation of new proposed reagents and instruments for use in LA testing, as compared to existing reagents/instruments as reference, which is a requirement of ISO15189 accreditation. That the existing reference was “fit for purpose” was already well established, as successful performance in regular EQA via the local EQA provider, the RCPAQAP.^13^ The study did not assess the “accuracy” of LA testing *per se*, for example based on clinical criteria. Further, we did not specifically assess the influence of anticoagulant therapy on LA test results in our study, with most anticoagulants potentially complicating LA detection and exclusion.^2^, ^12^ As a strength, our study data (Table 2) comprised the recommended 120 normal individuals suggested by the ISTH SSC,^2^ and is probably larger than that used by most laboratories attempting to verify NRRs for LA testing. Moreover, according to both ISTH SSC^2^ and CLSI,^7^, ^16^ a smaller number of normal individuals can be used if verification of the manufacturer's NRR or cutoff is the aim, with as few as 20 samples potentially being sufficient according to CLSI.^16^ Utilizing our data, and in view of other findings, we could feasibly adopt a cutoff of 1.2 for the dRVVT screen/confirm normalized ratio, as recommended by the manufacturer, and used by most other laboratories in Australia,^13^ and as based on CLSI guidance,^16^ with ≥90% of local values being within the manufacturer recommended limits. Alternatively, we could feasibly adopt a cutoff of 1.3, based on statistical findings with 2.5th–97.5th percentiles (Table 2). Ultimately, such cutoffs reflect a trade‐off between sensitivity and specificity, with too low a cutoff capturing potentially LA‐negative patients, and too high a cutoff missing potentially LA‐positive patients.

## 5 CONCLUSIONS

We essentially verified the utility of the HemosIL dRVVT‐based assay for LA on ACL TOPs and can confirm that these assays have been evaluated as “fit for purpose,” a terminology employed within the ISO15819 standard. We plan to adopt the Cephen reagents for use as paired APTT reagents on the ACL TOPs. Our evaluation identified good performance of these reagents compared to existing methods. Moreover, the APTT reagents represent open methods able to be adapted to a wide variety of instrument platforms, naturally including the ACL TOPs. We also essentially largely verified the manufacturer NRRs for LA testing. We will continue to monitor our LA testing, and performance in EQA, to decide if an additional (e.g., clinical) study is required to further verify cutoffs in use.

We also recognize that accreditation requirements differ according to geographical locality. In Australia, laboratories need to be accredited to ISO 15189 standard. Accreditation of Australian laboratories is overseen by NATA, the National Association of Testing Authorities, and also requires that laboratories adhere to a set of standards as outlined by various guideline documents issued by NPAAC, the National Pathology Accreditation Advisory Council.^24^, ^25^ Thus, not all the requirements that Australian laboratories need to fulfill for accreditation will be identical to those required to be fulfilled by laboratories in other geographical locations and vice versa. Nevertheless, we feel that our experience as outlined here, will still provide a useful guide to other laboratories intending to evaluate the ACL TOP 50 Family for use in their laboratories or networks, even if different accreditation requirements apply, and especially if they desire to standardize or harmonize tests and test procedures.

Finally, this evaluation of LA methods on the ACL TOP 50 Family (550 and 750 in this report) of instruments will enable harmonization of LA testing across all laboratories that perform such testing in our network, being the largest public pathology network in Australia. The ACL TOP 50 Family instruments are now currently installed throughout our network for accredited assays. Universal standard operating procedures (SOPs) and competency and training documents are also in preparation and evaluations for other specialized assays are also in progress.

## CONFLICT OF INTEREST

The authors have no conflicts of interest to disclose.

## Supporting information

Fig S1

Fig S2

Fig S3

Fig S4

## Data Availability

The data that support the findings of this study are available on request from the corresponding author.
