# Supplementary figures and images for: A multi‐laboratory assessment of lupus anticoagulant assays performed on the ACL TOP 50 family for harmonized testing in a large laboratory network
Source: Int J Lab Hematol. 2022 Mar 1;44(3):654–65. doi: 10.1111/ijlh.13818 (PMC9311435; doi:10.1111/ijlh.13818)

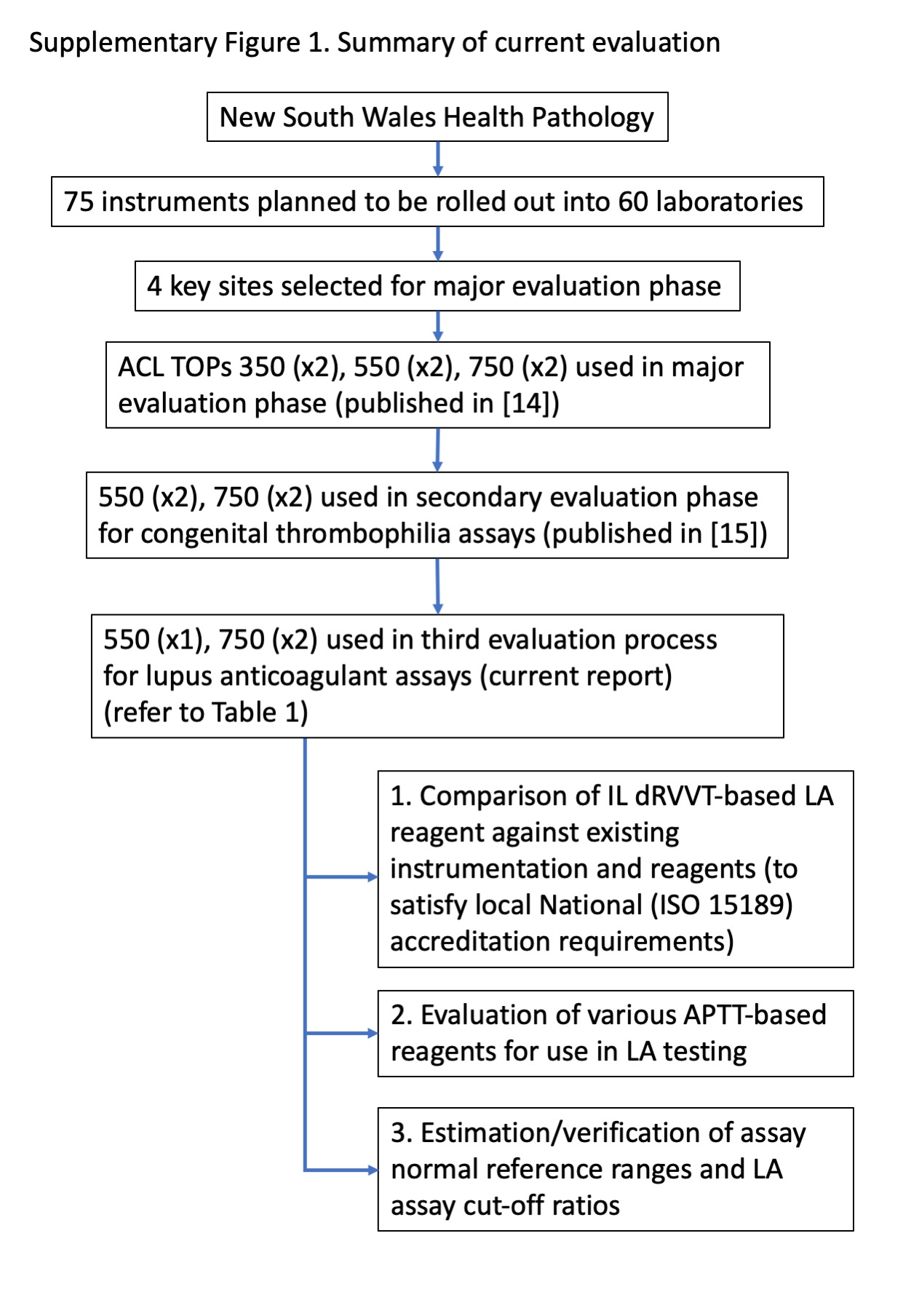

Supplement: Supplementary file 1 — Fig S1 [file IJLH-44-654-s002.jpg]

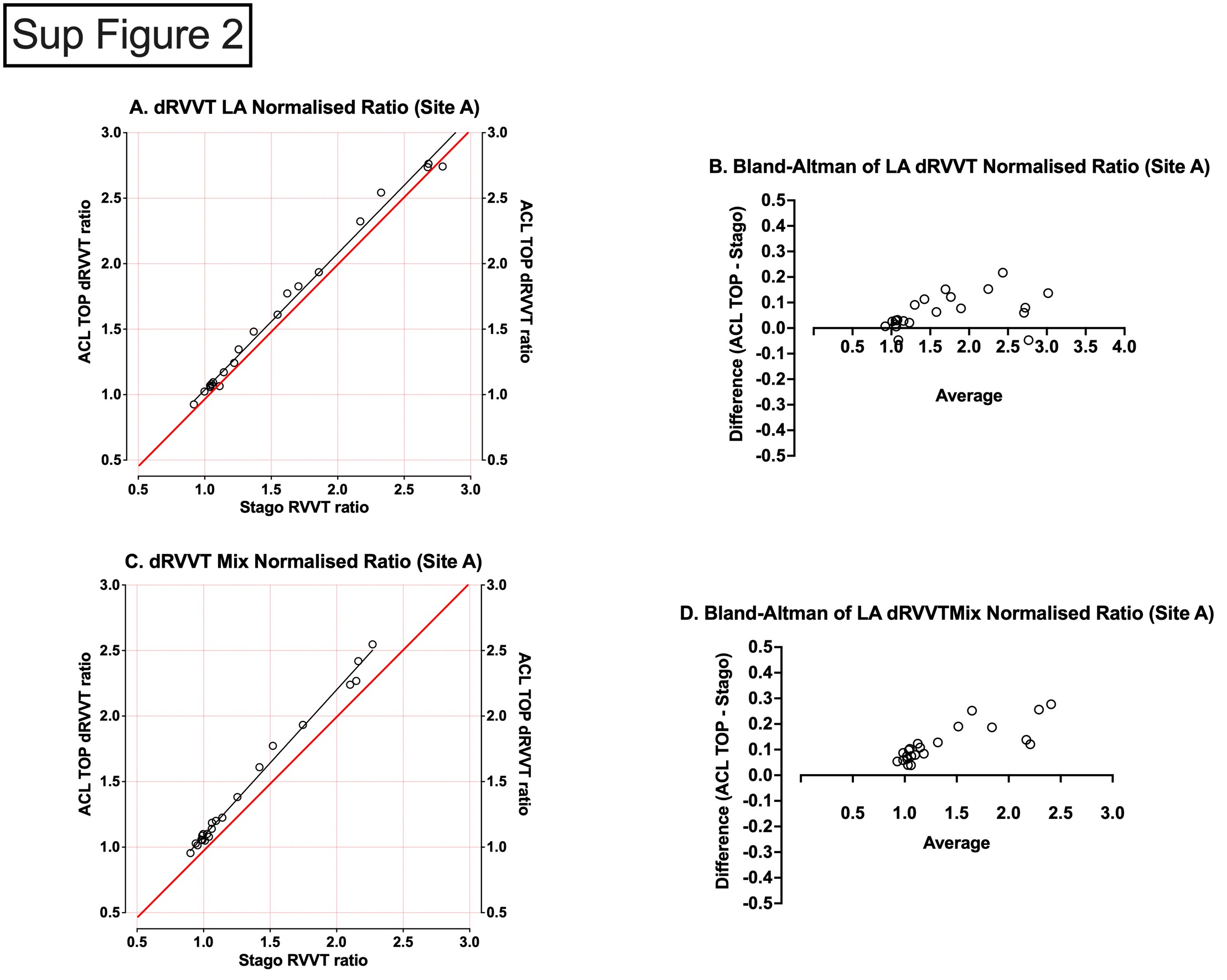

Supplement: Supplementary file 2 — Fig S2 [file IJLH-44-654-s004.jpg]

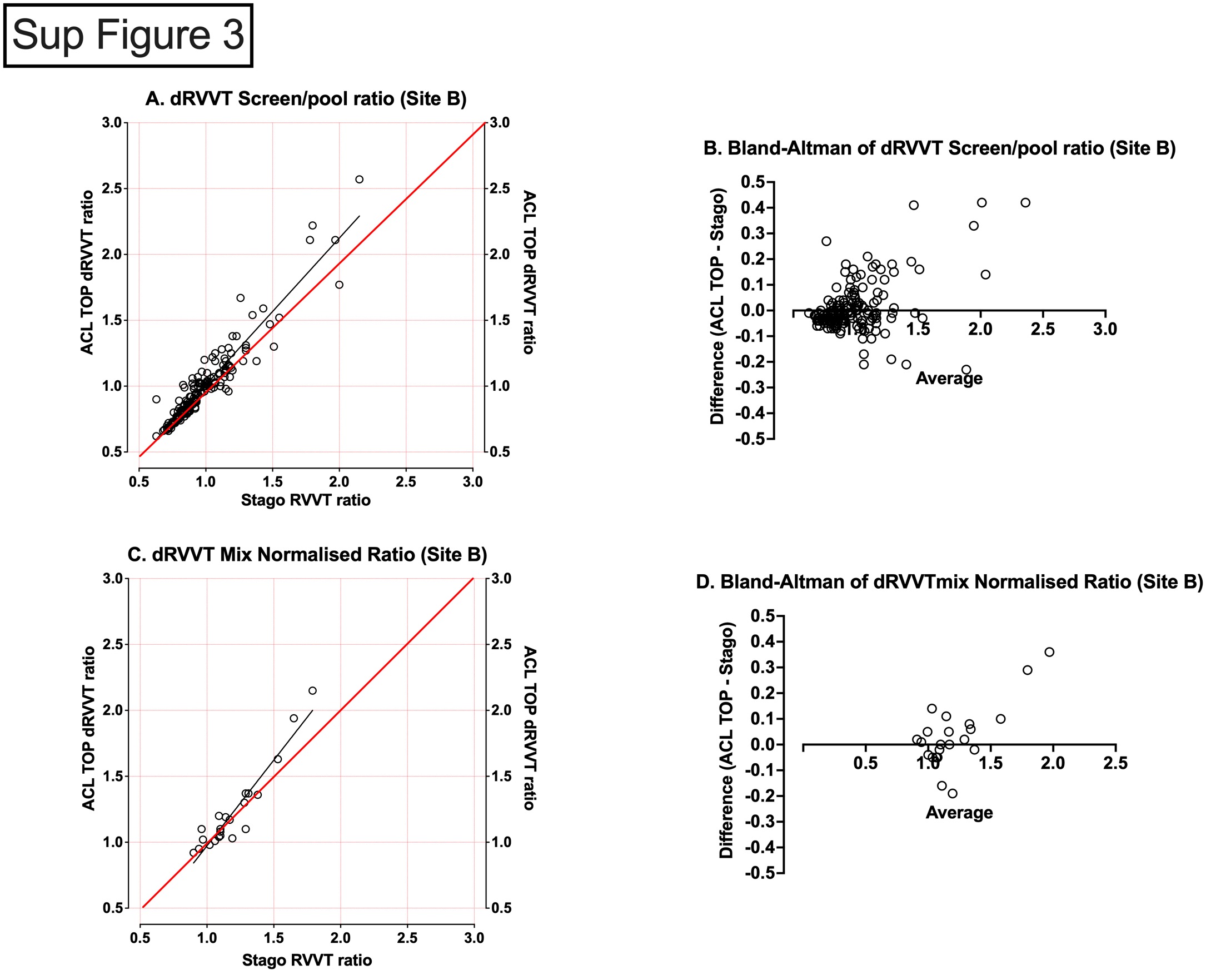

Supplement: Supplementary file 3 — Fig S3 [file IJLH-44-654-s003.jpg]

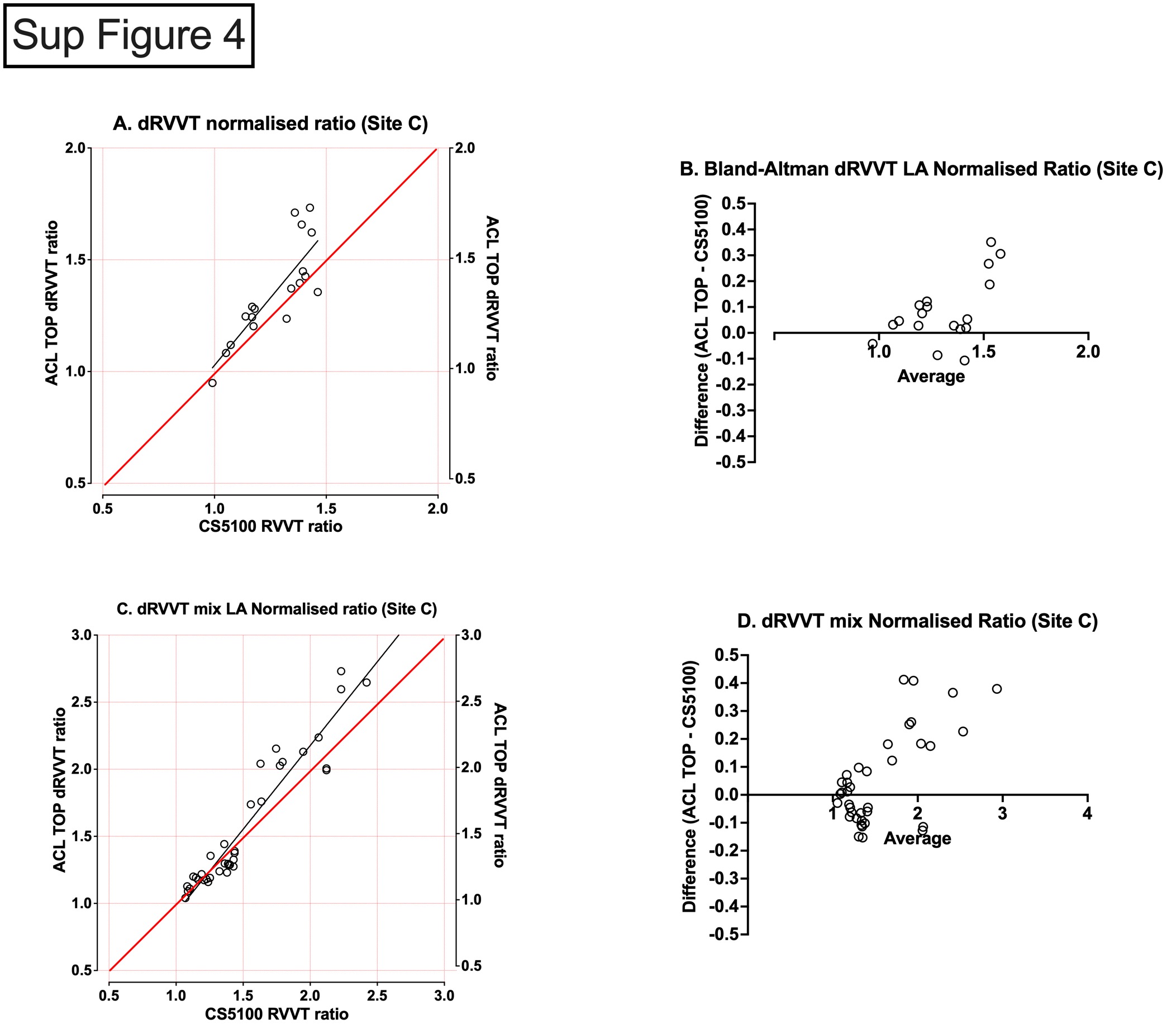

Supplement: Supplementary file 4 — Fig S4 [file IJLH-44-654-s001.jpg]
